# Ancient Mitochondrial Genomes Provide New Clues in the History of the Akhal-Teke Horse in China

**DOI:** 10.3390/genes15060790

**Published:** 2024-06-15

**Authors:** Siqi Zhu, Naifan Zhang, Jie Zhang, Xinyue Shao, Yaqi Guo, Dawei Cai

**Affiliations:** 1 Department of Archaeology, School of History, Wuhan University, Wuhan 430072, China; sqz0830@163.com; 2 Research Center for Chinese Frontier Archaeology of Jilin University, Changchun 130012, China; zhangnf19@gmail.com (N.Z.); 15764300208@163.com (Y.G.); 3 National Centre for Archaeology, Beijing 100013, China; 4 Xinjiang Institute of Cultural Relics and Archaeology, Ürümqi 830011, China; nxj198417@gmail.com; 5 Department of Archaeology, University of Southampton, Avenue Campus, Southampton SO17 1BF, UK; xs1v22@soton.ac.uk

**Keywords:** ancient DNA, Akhal-Teke, Shihuyao tombs, Silk Road, mitochondrial DNA

## Abstract

This study analyzed ancient DNA from the remains of horses unearthed from the Shihuyao tombs. These were found to date from the Han and Tang Dynasties in Xinjiang (approximately 2200 to 1100 years ago). Two high-quality mitochondrial genomes were acquired and analyzed using next-generation sequencing. The genomes were split into two maternal haplogroups, B and D, according to a study that included ancient and contemporary samples from Eurasia. A close genetic affinity was observed between the horse of the Tang Dynasty and Akhal-Teke horses according to the primitive horse haplotype G1. Historical evidence suggests that the ancient Silk Road had a vital role in their dissemination. Additionally, the matrilineal history of the Akhal-Teke horse was accessed and suggested that the early domestication of the breed was for military purposes.

## 1. Introduction

There are approximately 600–1000 horse breeds in the world today, with the Akhal-Teke representing one of the oldest [1]. Documentation suggests that Akhal-Teke horses have been bred for over 3000 years. The breed is native to Turkmenistan in Central Asia and was initially used for war and riding [2]. These horses are known for their speed, durability, and distinct metallic hair traits (Figure S1). Many notable horse breeds, including Thoroughbreds, have Akhal-Teke ancestry [3].

The earliest record of the Akhal-Teke in Chinese history was 2100 years ago during the Western Han Dynasty. It was called the Dayuan horse (or Ferghana horse) in ancient China. After the Han–Dayuan war, Emperor Wu, who was fond of horses, ordered the soldiers to bring over 1000 Dayuan horses to the Central Plains, and the emperor named them ‘heavenly horses’. It was reported that Emperor Wu crossbred Akhal-Teke and other horses with Mongolian horses [4,5]. Subsequently, the productivity and military hardware in the Han Dynasty also increased considerably. The administration of the Western Han Dynasty established the district government and seized control of the Western Regions in 60 CE [4,5]. The Central Plains dynasty and the Western nations were also engaged in several political, economic, and cultural contacts [6]. This resulted in multiple introductions of the Akhal-Teke from Central Asia into China. Furthermore, the splendor of the Central Plains Empire can be observed in the imperial artwork of the Han and Tang dynasties, such as the golden horse of Maoling, the Hetian Jade horse of Pingling, and the Night-Shining White painting by Han Gan (Figure S2).

The Akhal-Teke thrived for thousands of years, from the Han to the Yuan Dynasty. It was used for marching and was considered a symbol of culture and spirit [7]. However, crossbreeding between Akhal-Teke and local horses was not successful, and because many of the Akhal-Tekes used in battle were castrated, these animals could not reproduce [8]. Furthermore, the Akhal-Teke is not well-suited to supporting weight. Armed cavalry wearing armor are more likely to choose powerful mounts. Since the robust Mongolian horse has no such drawbacks, the stallions imported from Central Asia died in large numbers [8]. These factors have all added to the mystique surrounding the history of the Akhal-Teke.

Analysis of the mitochondrial DNA of contemporary horses has recently gained increasing international attention. This has advanced rapidly from analysis of the D-loop region [1,9,10] to genome-wide mitochondrial research [11,12,13], and has shown that the horses, including Akhal-Teke, have high levels of mitochondrial genetic variation [14]. A previous study identified a G1 haplotype unique to the Akhal-Teke breed [1]. However, the number of samples is extremely limited. The study of ancient horses in terms of population migration and cultural interchange has gained increasing popularity due to both archaeological discoveries and resources and advances in DNA sequencing technology (especially next-generation sequencing). Akhal-Tekes have been present in China since the Han Dynasty, and molecular archaeological research will help understand the domestication and dissemination of this breed. This investigation performed a mitochondrial genome-wide analysis of the remains of two ancient horses unearthed from the Shihuyao tombs of the Han and Tang Dynasties in Xinjiang. One of these horses had a maternal genetic relationship with the Akhal-Teke. Using a combination of the literature and archaeological evidence, the history of the Akhal-Teke in ancient China could be traced, revealing the history of ancient Eastern and Western cultural exchanges.

## 2. Material and Methods

### 2.1. Shihuyao Tombs

**Figure 1:**
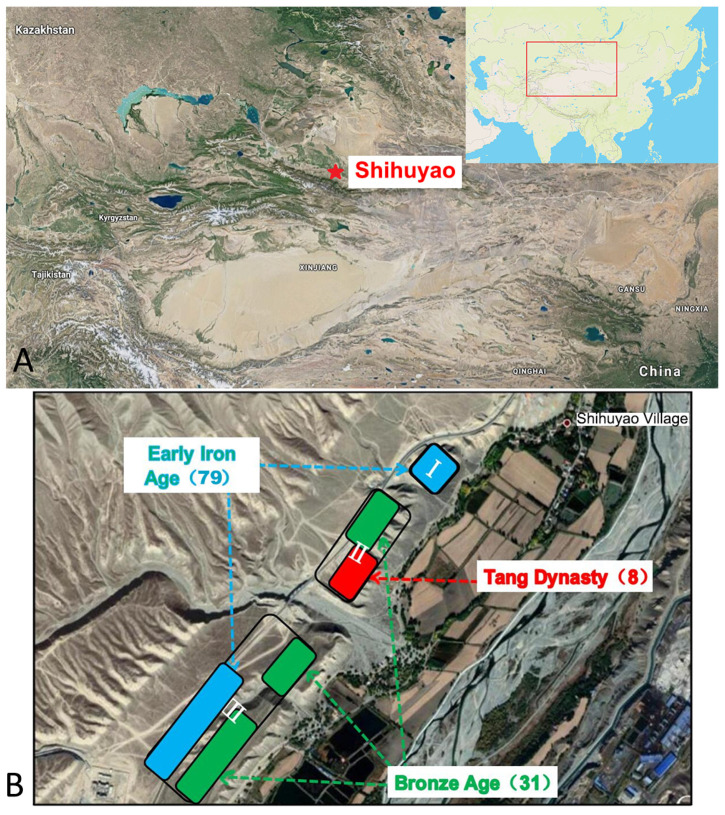
Satellite image map showing (**A**) the geographical location and (**B**) excavation of the Shihuyao tombs. The numbers in brackets represent the number of tombs.

Tombs I, II, and III in Shihuyao Village are located at 44.09° N, 86.05° E, northwest of the Manas River, on the northern foothills of the Yilan Habir Mountain (Figure 1). This is situated on the southern edge of the Junggar Basin where there is a mountain oasis that receives water from snowmelt and glaciers and is a haven of light and heat. This has long been an ideal location for animal husbandry, as well as horse domestication. Ili Autonomous Prefecture, a well-known horse-producing region in Chinese history, is located a few hundred kilometers west of the site. The horses of that area and the Akhal-Tekes of Dayuan were formerly called “Tianma” during the reign of Emperor Wu of the Han Dynasty [4]. Furthermore, it is on record that Ili horses have Akhal-Teke blood [15]. Additionally, due to its proximity to the Silk Road, the region received a continuous influx of various species, such as the grape, from the West throughout the Han and Tang dynasties [4].

The excavation site extends over 6000 m^2^ and includes 118 dispersed graves, of which 31 are Bronze Age tombs from the Andronovo culture, 79 date from the early Iron Age, and 8 tombs date from the Tang Dynasty (Figure 1). Over 250 pieces (groups) of artifacts have been unearthed from the Shihuyao tombs, including pottery, metal, wood, bone tools, and jade items. To date, the tombs from the Andronovo culture at the Shihuyao site in Shihezi City have provided the largest and richest set of artifacts unearthed in the Tianshan District of central Xinjiang [16]. However, there have been few studies on the tombs from the Han and Tang Dynasties. Horse bones were buried at the sites of tombs II and III, associated with the rich decorations characteristic of tombs of the Tang Dynasty. The head of a horse was found in the southeastern corner of the tomb, together with limbs but no vertebrae.

### 2.2. Preparation of Bone Samples

**Figure 2:**
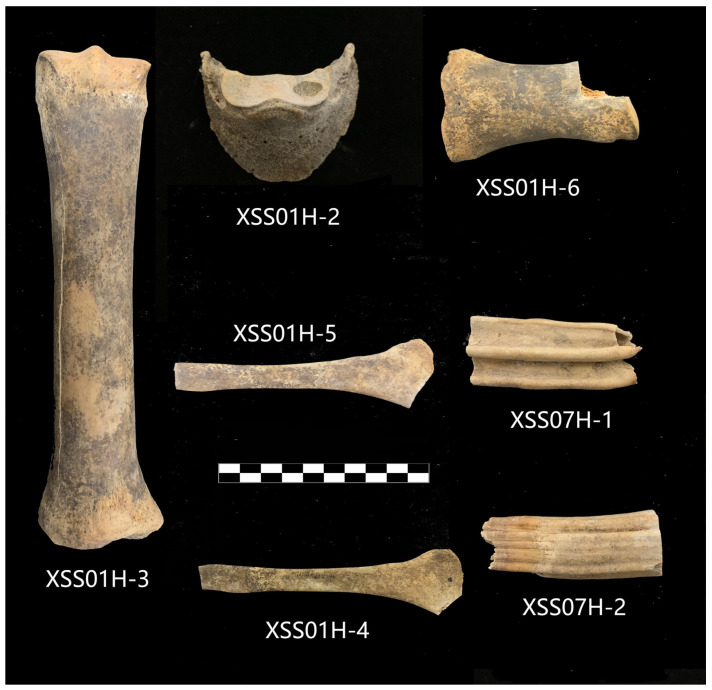
The ancient samples used in this study.

Eight samples were collected from the two sets of remains discovered in the Shihuyao tombs (XSS) and were morphologically identified as equines. Since the climate of the local area is hot, with high temperatures and low rainfall, the samples were well preserved (Figure 2). Of these, six samples (XSS01H) were acquired from the tomb of the Western Han Dynasty (206 BCE–24 CE) and the remaining two (XSS07H) were from the Tang Dynasty tomb (618–907 CE). The funerary wares unearthed in the tombs were also dated. XSS01H (NO. BA230588) was sent to Beijing University for radiocarbon dating, showing that these dated to 389–208 cal BCE (calibrated two-sigma, IntCal20, OxCal v4.4.4 Bronk Ramsey).

### 2.3. DNA Extraction and Sequencing

The dust and clay on the outer surfaces of the teeth or bones were cleaned with a fur brush. The cleaned samples were then cut into smaller pieces, soaked in 10% bleach for 20 min, rinsed with ethanol and distilled water, and UV-irradiated for 30 min on each side. Then, with the help of a hand drill, the samples were ground into a powder from which the DNA was extracted using a modified silica spin column method [17] in a dedicated ancient DNA laboratory at Jilin University. Briefly, the samples were decalcified by the addition of 200 mg of powdered sample in 3.9 mL of 0.465 mol/L EDTA, followed by incubation at 4 °C for 12 h. This was followed by the treatment of the mixture with 0.1 mL of 0.4 mg/mL proteinase K in a rotating hybridization oven at 50 °C (220 rpm) overnight, after which the supernatant was transferred into an Amicon^®^ Ultra-4 centrifugal filter device (Merck Millipore Ltd., Darmstadt, Germany, 10,000 nominal molecular weight limit), reduced to less than 100 µL, and purified using a QIAquick^®^ PCR Purification Kit (Qiagen, Hilden, Germany), according to the instructions. 

DNA libraries were prepared using a NEBNext^®^ UltraII™ DNA Library Prep Kit for Illumina^®^ (New England Biolabs, Ipswich, MA, USA). Specifically, the extracted DNA (50 µL) was end-repaired and A-tailed by adding 7 μL of NEBNext Ultra II End Prep Reaction Buffer and 3 μL of NEBNext Ultra II End Prep Enzyme Mix, and incubated for 40 min at 20 °C and then for 30 min at 65 °C. The adaptor was ligated to the dA-tailed DNA fragments by the addition of 30 µL of NEBNext Ultra II Ligation Master Mix, 1 µL of NEBNext Ligation Enhancer, and 2.5 µL of NEBNext Adaptor for Illumina (dilution 1:10), and incubated for 20 min at 20 °C. The adaptor was then linearized by adding 3 µL of USER Enzyme followed by incubation for 15 min at 37 °C. The adaptor-ligated DNA was cleaned without size selection using a MinElute PCR Purification Kit (Qiagen), following the instructions provided by the manufacturer. PCR enrichment was performed by using 30 µL of NEBNext Ultra II Q5 Master Mix, 1 µL of Index Primer, 1 µL of Universal PCR Primer, and 18 µL of adaptor-ligated DNA. The PCR cycling conditions comprised an initial denaturation at 98 °C for 30 s, 14–16 cycles of 98 °C for 10 s, 65 °C for 75 s, and a final extension at 65 °C for 5 min. The PCR products were then purified using an Agencourt AMPure XP Nucleic Acid Purification Kit. All the bar-coded libraries were sequenced on an Illumina HiSeq X Ten platform (paired-end 150 bp).

### 2.4. Data Analysis

The sequencing reads were processed and aligned against the horse reference genome EquCab3.0 [18] via the PALEOMIX pipeline [19] with default parameters and the recommendations from previous research [20]. The seeding was disabled. Briefly, paired-end reads longer than 25 nucleotides were trimmed with AdapterRemoval v2.2 [21] and aligned against the reference genomes using BWA [22], retaining alignments with mapping qualities superior to 25. PCR duplicates were removed using Picard MarkDuplicates. Finally, all ancient and modern reads were locally realigned around indels using GATK [23]. Postmortem DNA damage was assessed via mapDamage2.0 [24], and to decrease the effect of post-mortem DNA damage, base quality rescaling and reads trimming were performed as previously described [25]. 

The clean reads were mapped against the mitochondrial genome (GenBank accession no. NC_001640), following the same procedure used for mapping against the nuclear genome. After the removal of duplicates, consensus mitochondrial sequences were generated using ANGSD [26] (-doFasta 2 -doCounts 1 -setMinDepth 3 -uniqueOnly 1 -remove_bads 1 -minQ 25 -minMapQ 25). Multiple alignments were performed with comparative mtDNA sequences from GenBank (Table S1) using MUSCLE v3.8.31 [27] with default parameters.

A whole-mitochondrial maximum-likelihood tree was reconstructed using 663 samples by RAxML-NG v.0.5.0 [28], and a median-joining network based on the D-loop region (sites: 15494–15740) of 63 individuals was constructed using Network10 (http://www.fluxus-engineering.com (accessed on 23 January 2024)). The TIM2+F+R5 substitution model was estimated by ModelFinder [29] and 1000 bootstrap pseudo-replicates were conducted to assess the robustness. Lastly, the tree was visualized online using the iTOL [30] website (https://itol.embl.de/ (accessed on 20 January 2024)) and the group information of the samples was color-coded. 

BEAST analyses were performed based on the Coalescent Constant Population and Bayesian Skyline demographic models via BEAST 2.5.1.0 [31]. The sequences were applied to the six partitions (first, second, and third codon positions, rRNA, tRNA, and control regions) by Partition Finder v2.1.1 [32]; however, the best substitution model was selected for each partition by a model generator, version 0.85 [33]. The substitution models applied to the six sequence partitions were the HKY+I+G model (first codon position = 3781 sites), the HKY+I+G model (second codon position = 3777 sites), the TrN+G model (third codon position = 3774 sites), the HKY+I+G model (transfer RNAs = 1580 sites), the HKY+I+G model (ribosomal RNAs = 2560 sites), and the TrN+I+G model (control region = 1204 sites). We calibrated the tree using tip dates. A relaxed molecular clock (log normal) was considered for each partition, 50,000,000 iterations were run, and samples were drawn every 10,000 steps. The final consensus tree was produced using TreeAnnotator 2.5.1.0 [34] and plotted with the Figtree v1.44 (http://tree.bio.ed.ac.uk/software/figtree/ (accessed on 2 June 2023)).

### 2.5. Authenticity of the Ancient DNA

All pre-PCR procedures were conducted in a dedicated ancient DNA laboratory that is physically separated from the post-PCR laboratory. Strict criteria for authenticating ancient DNA sequences were applied [35]. Working areas and benches were frequently cleaned with bleach and exposed to UV radiation to remove potential contaminant DNA. All workers wore protective clothing, specifically, coveralls with hoods, facemasks, and gloves. To ensure the reproducibility of the results, at least two independent DNA extractions and multiple PCR amplifications were carried out for each sample. To detect any possible contamination, blank controls were included in each DNA extraction and PCR setup. Postmortem DNA damage of six samples was determined with mapDamage2.0 [24] and was consistent with the characteristics of ancient DNA (Figure S3). 

## 3. Results

### 3.1. Sequencing Results

Six DNA libraries were successfully acquired from eight samples from the two sets of remains (Table 1), and they were identified as horses. A comparison of the X chromosome and autosomal coverage revealed that the samples were derived from one male and one female. An average mtDNA depth of coverage >25 was identified. The sequencing data acquired from this investigation have been deposited in GenBank with accession numbers OR067839 and OR067840.

### 3.2. Nucleotide Positions of the Akhal-Teke

The mitochondrial haplotypes of 11 primitive horses have been identified based on the mitochondrial D-loop region, showing that the G1 haplotype is unique to the Akhal-Teke breed [1]. Therefore, based on comparisons with the sequence of the G1 haplotype, the sequence variations of 21 samples are shown in Table 2, including XSS07H, 15 Akhal-Teke horses, and 5 horses with the same variable nucleotide positions as the G1 haplotype. The present study found variable nucleotide positions at 15495-C, 15521-A, 15596-G, 15602-T, and 15720-A. When these were compared with the ancient DNA sequences, surprisingly, XSS07H matched the nucleotide positions of the G1 haplotype perfectly (Table 2). This suggests that horses with the primitive maternal haplotypes of the Akhal-Teke were present along the ancient Silk Road in China at least 1100 years ago. Furthermore, two Thoroughbreds also exhibited the G1 haplotype, consistent with the current belief that Akhal-Tekes contributed genetically to Thoroughbreds [3].

### 3.3. Phylogenetic Trees

**Figure 3:**
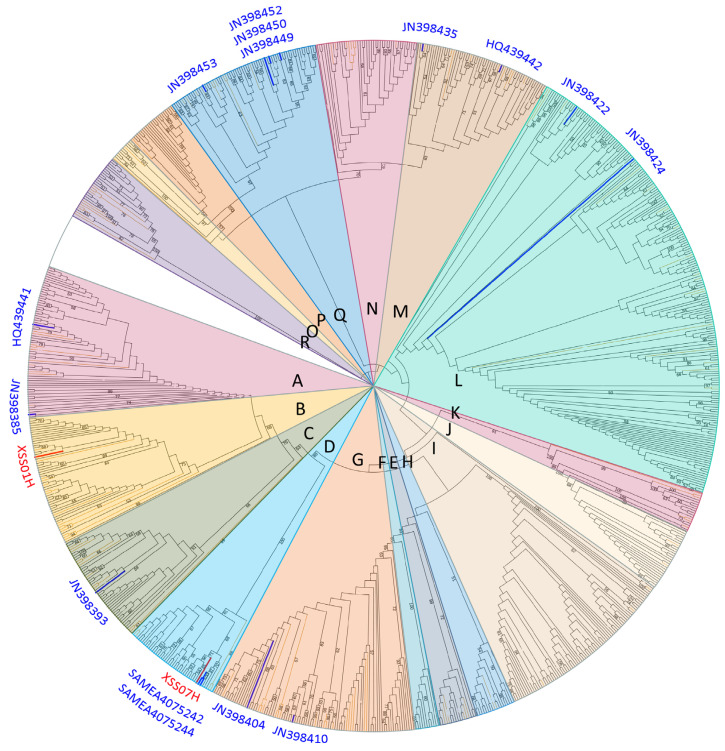
The maximum likelihood tree of 663 horse samples. The red and blue branches indicate the XSS and Akhal-Teke samples, respectively. The yellow branches indicate the ancient samples.

From the mitochondrial genome-wide maximum likelihood tree, the phylogenetic associations of the ancient samples (Figure 3) show the consistency of the phylogenetic analysis using the Bayesian framework (Figure S4). The phylogenetic tree shows the division of XSS horses into two branches. According to previous studies on mitochondrial haplogroups in horses [11], these are associated with haplogroup B (XSS01H buried in the Western Han Tomb) and D (XSS07H buried in the Tang Tomb), respectively. This suggests that the maternal origins of the horses from the two periods are different. Interestingly, XSS07H and seven Jeju horse samples were adjacent to the two contemporary Akhal-Teke samples (SAMEA4075242 and SAMEA4075244), indicating a close maternal genetic link. 

### 3.4. Median-Joining Network Analysis

**Figure 4:**
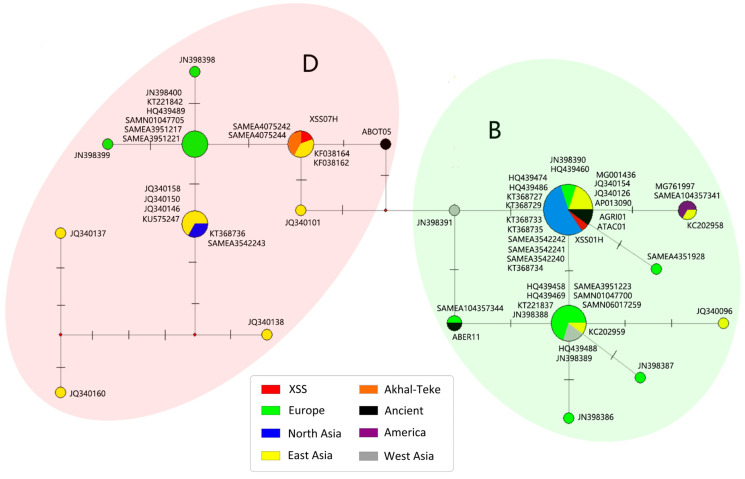
Median-joining network of the mitochondrial D-loop region of the two XSS samples together with 61 samples belonging to haplogroups B and D.

Given that the mitochondrial sequences of the two ancient horses belonged to haplogroups B and D, a median-joining network was constructed for a clearer illustration of the maternal genetic relationships (Figure 4).

It can be seen that the XSS horses are divided into two major groups according to the mitochondrial D-loop region, consistent with the analysis of the complete mitochondrial sequences. Haplogroup B was found to comprise two main haplotypes, whereas haplogroup D was more complex. XSS01H shared the same central haplotype with 10 North Asian, 4 East Asian, 2 ancient, and 2 European horses. Two Akhal-Teke horses and two modern Jeju horses from East Asia had the same haplotype as XSS07H.

### 3.5. Akhal-Teke Bayesian Skyline Demographic Profile

**Figure 5:**
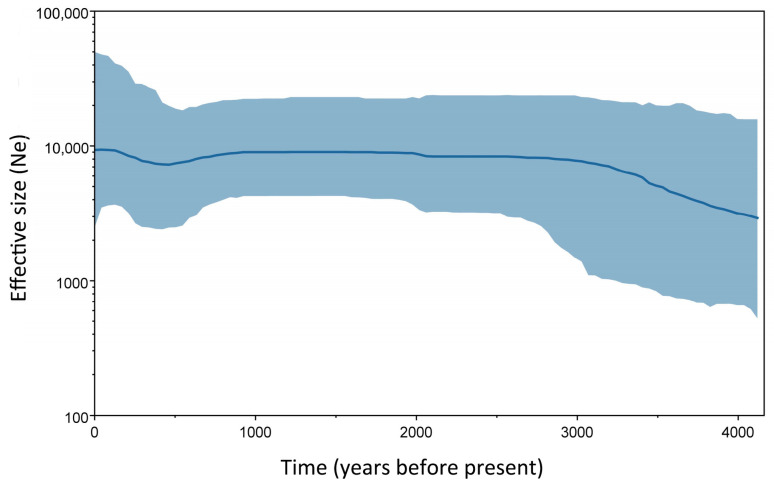
Bayesian skyline plot analysis of 16 Akhal-Teke samples. The graph indicates the population history using the median and 95% HPD intervals. The *x*-axis represents the age (YBP), and the *y*-axis represents the effective population size.

Based on the whole mitochondrial genome, 16 Akhal-Teke samples were used to infer the maternal population history using a Bayesian skyline plot (BSP) (Figure 5). This indicated that the effective population size (*Ne*) of the breeds has been expanding slowly since the modern domestic horses expanded rapidly across Eurasia from about 2000 BCE [13] and has been stable with minimal variation since 2600 years before present (YBP).

## 4. Discussion

### 4.1. Maternal Lineage Analysis of XSS Horses

The representative Andronovo culture found in the Shihuyao tombs can be traced back to the Bronze Age [16]. These fairly common traditional mound-covered graves have a long history and have persisted since the late Han and Tang dynasties, despite some changes in the excavated burial items. This suggests that the local population remained essentially constant without noticeable replacement and that the prairie nomad culture was always dominant. The custom of burying horses, as seen in the Shihuyao tombs, began to appear during the early Iron Age, although this was relatively rare and was only in senior tombs. It was also discovered that only women were buried in the tombs with horses, reflecting the unique status of female aristocrats (Table 1). 

Considering the maternal haplogroup B horses, the two central haplotypes correspond to the eastern and western lineages, respectively. These represent the northern and eastern Asian horse and the European horse (Figure 4), consistent with the finding that haplogroup B has two main branches based on mitochondrial genome-wide phylogenetic analysis (Figure 3). It can be seen that the Han Dynasty horse (XSS01H), which belongs to the eastern lineage, shared the center haplotype with two other ancient horses (ATAC01, discovered in Bronze Age Georgia, and AGRI01, discovered in medieval Kazakhstan) which are geographically closely related to modern Asian horses, indicating that the eastern lineage of haplogroup B has a long history.

In terms of horses with the primitive maternal Akhal-Teke haplotype, XSS07H was found to share the same lineage as the two Akhal-Teke (SAMEA4075242 and SAMEA4075244) and the two Jeju (KF038162 and KF038164) horses (Figure 4). The two Jeju horses had a maternal haplotype related to the Akhal-Teke due to recent crossbreeding and their unique historical background. Jeju is an island known for horse breeding because of the unique local environment and the presence of fewer grazing animals. The Mongols took over the island after the Goryeo–Mongolian War (1231–1273 CE), and they bred horses there [36]. Various horses, including those with Akhal-Teke ancestry, were transported from China to Jeju Island [37].

### 4.2. The Significance of the Silk Road in the Spread of Ancient Horses

The selection of a horse is symbolic, whether it represents a religious sacrifice, a longing for intimacy, or prestige and riches [38]. It was found that the funerary horse from the Han tombs belonged to mitochondrial haplogroup B, whereas that from the Tang tombs belonged to mitochondrial haplogroup D. Although it is impossible to infer changes in local breeds due to the small number of sequenced samples, it has been shown that there was communication between different populations during the period. Instead of horses belonging to haplogroup B, horses belonging to haplogroup D with excellent traits, such as Akhal-Teke, were highly favored by Tang Dynasty nobles and were used in funerals. The priceless ornaments discovered from the tombs of aristocrats also confirm frequent international exchanges. The most well-known route of communication between the East and the West was the Silk Road.

In terms of the history of the Silk Road, the eastern (from Chang’an, Luoyang to Yumen Pass, Yang Pass) and middle (from west of Yumen Pass, Yangguan to Cong Ling Ridges) sections were opened during the Western Han Dynasty [39]. However, only the middle and south trails were accessible in the middle section. The location of the Shihuyao tombs was not in the corridor connecting East and West at the time. However, during the Eastern Han Dynasty, the middle section provided a new northern route that crossed through the Shihuyao tombs and the area where the Akhal-Tekes originated (from Dunhuang via Yiwu [Hami], Pulei Sea [Balikun Lake], Beiting [Jimsar], Luntai [Banquan], Gongyue City [Huocheng], and Suiye [Tokmark] to Talas) [39]. This route was an important channel throughout the Sui and Tang Dynasties. Different animal species spread throughout this route as a result of diverse human activities. A previous study revealed that there were extensive exchanges of genetic information among *C. bactrianus* populations in regions along the Silk Road [40]. It can be speculated that the Akhal-Tekes and their descendants, who shared maternal ancestry with XSS07H, were probably imported from Central Asia in this way. Another speculation would be that the G1 haplotype can represent wild types that were once ubiquitous and separately introgressed through local wild populations.

### 4.3. Analysis of the Maternal Demographic History of the Akhal-Teke

The Akhal-Teke has over 3000 years of breeding history [2]. The BSP analysis in the present investigation shows that the female lineages of the Akhal-Tekes grew annually from approximately 4200 to 2600 years ago, a period when large numbers of horses were domesticated for military purposes. This was seen in the region of origin and the neighboring ethnic groups, such as the Zhou and Hun, who actively introduced and bred horses during this period. It was recorded that Akhal-Tekes were regarded as the essential mount for the Huns during the early Western Han Dynasty’s War of Baiden, long before Emperor Hanwu [4,5]. However, the effective population size has remained essentially the same overall for the last 2600 years, indicating that the maternal population has not undergone further selection, consistent with the findings of previous studies that a single patriline was chosen for the breeding of horses, while multiple matrilines existed [11].

## 5. Conclusions

In this investigation, ancient DNA from the remains of two horses unearthed from the Shihuyao tombs was examined using next-generation sequencing methods. This study represents the first identification of the original nucleotide position of the Akhal-Teke horses in ancient horses from China. A possible dispersal route of Akhal-Teke maternal lineages was also discovered, supported by the history of extensive trade and cultural exchanges along the Silk Road. Furthermore, analysis of the maternal demographics of the Akhal-Teke showed an annual growth from approximately 4200 to 2600 years ago. This suggests that the demand for military horses played an important role in the early domestication of the Akhal-Teke in this particular historical context. Since the Akhal-Teke has historical significance both as a saddle horse and cultural symbol, more samples are needed for future analyses to verify these results.

## Figures and Tables

**Table 1:** Sample information.

Lab Code	Arch Code	Element	Period	Sanger Sequencing	DNA Library Construction	Raw Reads	Mapped Reads	Endogenous DNA	X Chromosome Coverage	Autosomal Coverage	Sex	Tomb Owner Sex	mtDNA Coverage
XSS01H-1	2017XSSIIIM54:1-4	os coronale (L)	Western Han Dynasty	Success	Success	89251214	14205018	31%	0.367	0.746	Male	Female	58.376
XSS01H-2	2017XSSIIIM54:1-5	pedal bone (L)	Western Han Dynasty	Success	Failure								
XSS01H-3	2017XSSIIIm54:1-35	metacarpal (L)	Western Han Dynasty	Success	Success	92467552	4640559	10%					
XSS01H-4	2017XSSIIIM54:1-42	hyoid bone (L)	Western Han Dynasty	Success	Success	33058070	5132236	31%					
XSS01H-5	2017XSSIIIM54:1-43	hyoid bone (R)	Western Han Dynasty	Success	Success	36194492	2405398	13%					
XSS01H-6	2017XSSIIIM54:1-1	pastern (L)	Western Han Dynasty	Success	Failure								
XSS07H-1	2017XSSIIM4:36	tooth	Tang Dynasty	Success	Success	31531568	3197891	20%	0.175	0.175	Female	Female	25.416
XSS07H-2	2017XSSIIM4:37	tooth	Tang Dynasty	Success	Success	46666564	5425248	22%					

**Table 2:** Haplogroups and sequence variations of the specimens related to the Akhal-Teke.

Sample	Variable Nucleotide Positions					Haplogroups	Origin
NC_001640	15495	15521	15596	15602	15720		
G1	C	A	G	T	A	D	Cieslak et al. [1]
XSS07H	C	A	G	T	A	D	This study
SAMEA4075242	C	A	G	T	A	D	Akhal-Teke
SAMEA4075244	C	A	G	T	A	D	Akhal-Teke
HQ439441.1	C	G	A	C	A	A	Akhal-Teke
JN398385.1	C	G	A	T	A	A	Akhal-Teke
HQ439442.1	C	G	A	T	A	M	Akhal-Teke
JN398435.1	C	G	A	T	A	M	Akhal-Teke
JN398393.1	C	G	A	T	A	C	Akhal-Teke
JN398404.1	C	G	A	T	A	G	Akhal-Teke
JN398410.1	C	G	A	T	A	G	Akhal-Teke
JN398422.1	C	G	A	T	A	L	Akhal-Teke
JN398424.1	C	G	A	T	A	L	Akhal-Teke
JN398449.1	C	G	A	T	A	Q	Akhal-Teke
JN398450.1	C	G	A	T	A	Q	Akhal-Teke
JN398452.1	C	G	A	T	A	Q	Akhal-Teke
JN398453.1	C	G	A	T	A	Q	Akhal-Teke
JQ340137	C	A	G	T	A	D	China
GolModII_Mon26_1999	C	A	G	T	A	D	Mongolia
KT221842	C	A	G	T	A	D	Thoroughbred
JN398398	C	A	G	T	A	D	Norwegian Fjord
Thoroughbred_thb13	C	A	G	T	A	D	Thoroughbred

## Data Availability

All novel sequences were deposited in GenBank under the accession numbers OR067839 and OR067840. Other bovid sequences were downloaded from the GenBank database (Supplementary Table S1).

## Supplementary Materials

The following supporting information can be downloaded at: https://www.mdpi.com/article/10.3390/genes15060790/s1, Figure S1: Akhal-Teke (Images by *Prof. Mortel*); Figure S2: (A) The golden horse of Maoling Mausoleum. (B) The Hetian Jade horse of Pingling Mausoleum. (C) Portrait of ‘Night-Shining White’ painted by HanGan; Figure S3: DNA damage patterns for ancient sample XSS01H. (A) Before rescaling and trimming and (B) after rescaling and trimming the region comprising the five first and last nucleotides sequenced. The red line shows C to T and the blue line shows G to A substitutions; Figure S4: The Bayesian phylogenetic tree of two XSS and 76 horse samples. For a clearer presentation of sample comparisons, 76 samples were selected from the Achilli et al. [11] study, which was the first to distinguish the A–R maternal haplogroups. The XSS specimens are shown in red. Bayesian posterior probabilities are provided next to each node; Table S1: The comparative mitochondrial DNA sequences from GenBank.
