# Supplementary figures and images for: Ancient Mitochondrial Genomes Provide New Clues in the History of the Akhal-Teke Horse in China
Source: Genes (Basel). 2024 Jun 15;15(6):790. doi: 10.3390/genes15060790 (PMC11203007; doi:10.3390/genes15060790)

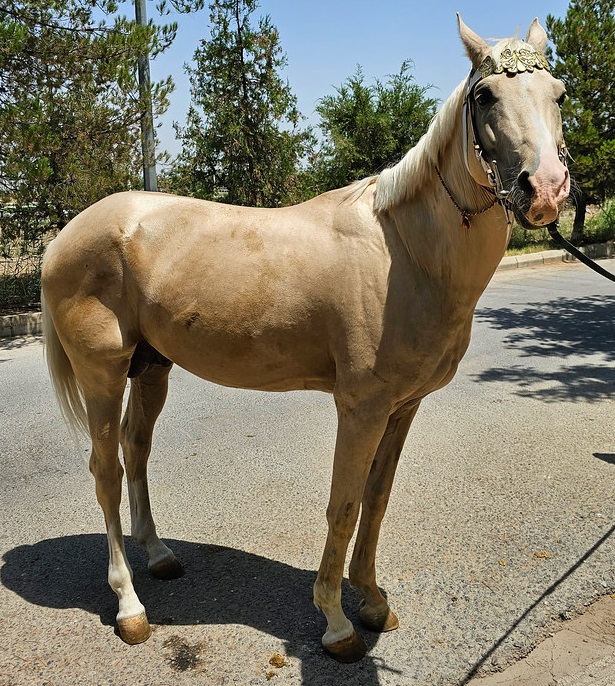

Supplement: Supplementary file 1 [file genes-15-00790-s001.zip › Figure S1.jpg]

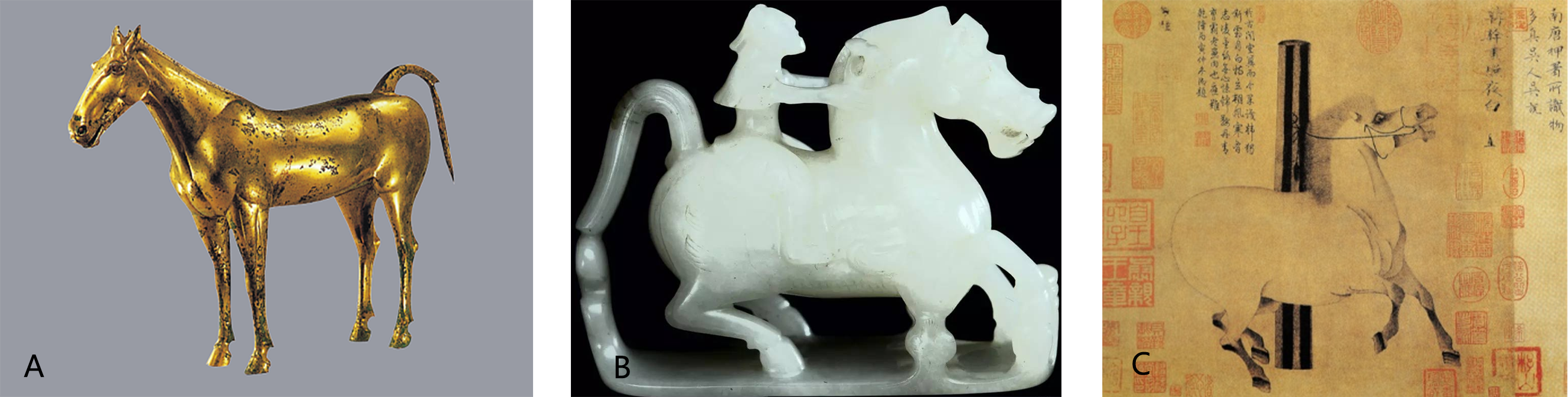

Supplement: Supplementary file 1 [file genes-15-00790-s001.zip › Figure S2.png]

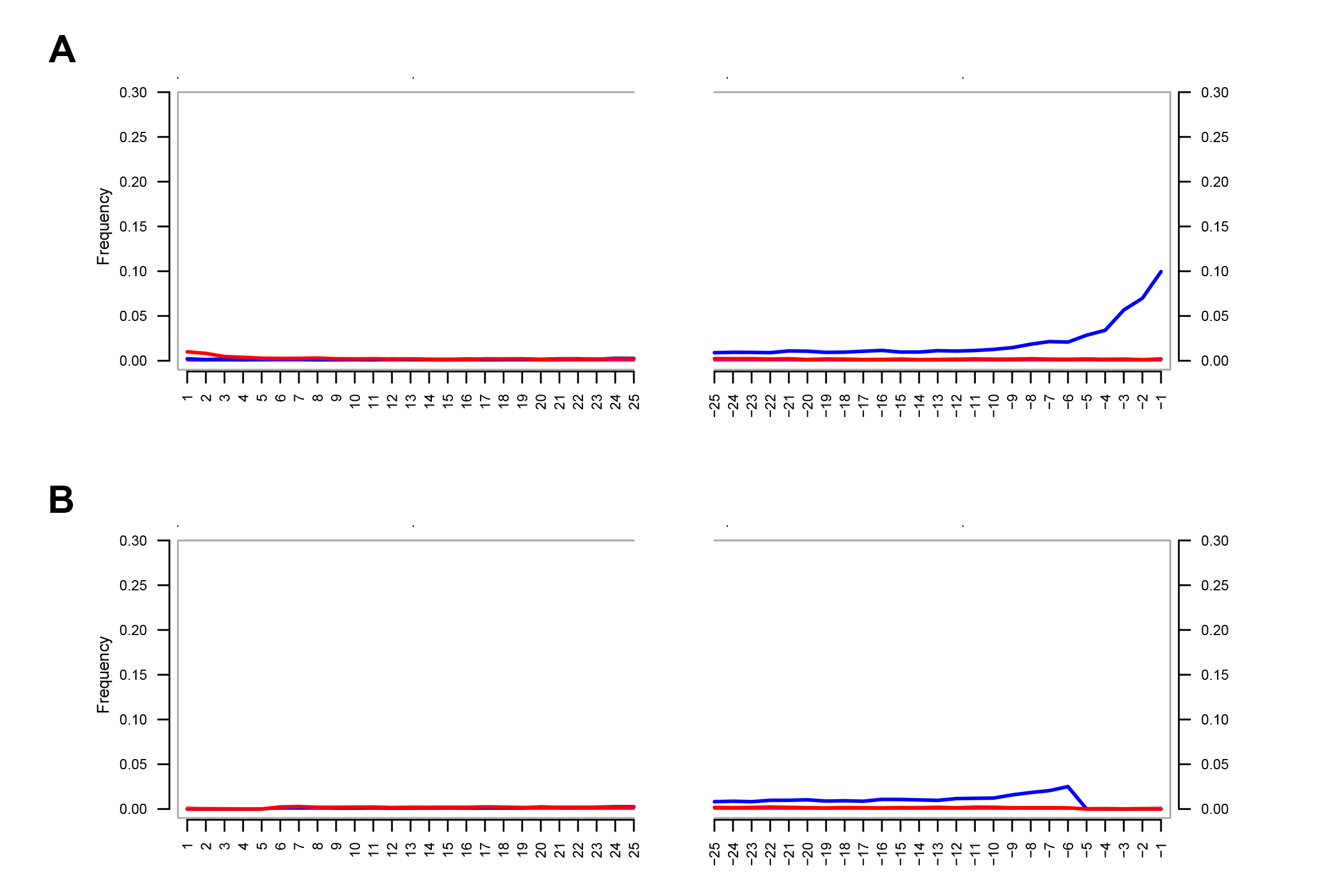

Supplement: Supplementary file 1 [file genes-15-00790-s001.zip › Figure S3.png]

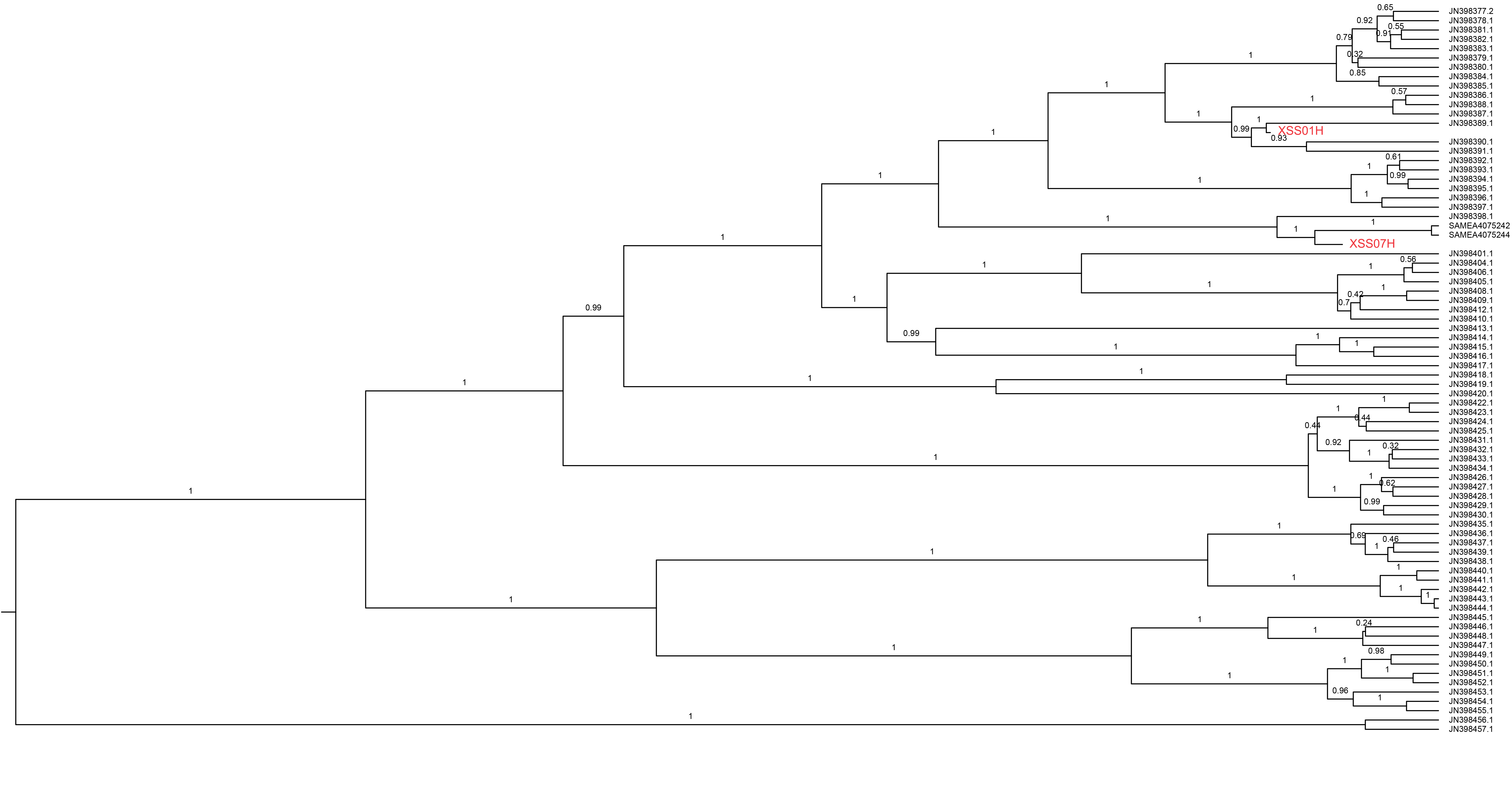

Supplement: Supplementary file 1 [file genes-15-00790-s001.zip › figure S4.png]
